# Recent Advances in Hydrogels for Tissue Engineering and Biomedical Therapeutics

**DOI:** 10.3390/gels11090733

**Published:** 2025-09-11

**Authors:** Hyun Jong Lee

**Affiliations:** School of Chemical, Biological and Battery Engineering, Gachon University, 1342 Seongnam-daero, Seongnam-si 13120, Republic of Korea; hjlee2@gachon.ac.kr

## 1. Introduction

Hydrogels represent a pivotal biomaterial platform that has fundamentally transformed approaches in tissue engineering and biomedical therapeutics. These three-dimensional polymeric networks, characterized by their ability to retain substantial amounts of water while maintaining structural integrity, possess unique physicochemical properties that closely mimic the native extracellular matrix (ECM) of biological tissues [1]. The inherent biocompatibility, tunable mechanical properties, and capacity for controlled drug release have positioned hydrogels as indispensable tools in regenerative medicine [2].

The clinical significance of hydrogels stems from their capacity to address critical challenges in tissue engineering, including the need for biocompatible scaffolds that support cellular proliferation, differentiation, and tissue integration. Traditional biomaterials often fail to adequately replicate the complex microenvironment required for optimal tissue regeneration, particularly in applications demanding sustained therapeutic agent delivery or precise mechanical property matching [3]. Hydrogels overcome these limitations through their versatile design capabilities, allowing for customization of porosity, degradation kinetics, and bioactive molecule incorporation [4].

Recent advances in hydrogel technology have expanded their applications across diverse biomedical domains, from bone tissue engineering requiring mechanically robust constructs to soft tissue applications demanding high flexibility and biointegration [5]. The development of composite hydrogels incorporating bioactive components such as hydroxyapatite for osteogenesis, growth factors for enhanced cellular responses, and antimicrobial agents for infection control has further broadened their therapeutic potential [6].

This Special Issue encompasses critical areas of hydrogel research, including bone tissue engineering applications utilizing hydroxyapatite–gelatin composites, advanced drug delivery systems employing carboxymethyl chitosan matrices, photo-crosslinkable gelatin methacrylate platforms for cartilage regeneration, and innovative microneedle systems for transdermal therapeutic delivery. Additionally, the collection addresses emerging applications in synthetic biology through giant unilamellar vesicle production, biotherapeutic delivery via electrostatic nanoparticle systems, advanced wound dressing technologies utilizing silk protein bilayers, and microbiome modulation strategies for infection control during tissue regeneration.

The multidisciplinary nature of hydrogel research necessitates integration of polymer science, materials engineering, cell biology, and clinical applications. This convergence has led to significant breakthroughs in understanding hydrogel–cell interactions, optimizing mechanical properties for specific tissue applications, and developing novel crosslinking strategies that enhance biocompatibility while maintaining structural integrity.

## 2. Overview of Papers Published in This Special Issue

This Special Issue, “Hydrogels for Tissue Engineering and Biomedical Therapeutics,” brings together eight research articles and two review papers highlighting recent advancements in hydrogel-based systems for biomedical applications. These contributions explore innovative synthesis methods, novel material properties, and diverse therapeutic applications spanning from tissue engineering to drug delivery systems.

The paper “Synthesis of Hydroxyapatite-Gelatin Composite Hydrogel for Bone Tissue Application” by Barrera Bernal et al. reports the development of a composite hydrogel using gelatin, di-amine polyethylene glycol, and genipin as a crosslinker with hydroxyapatite incorporation. The hydrogels demonstrated elastic modules and mechanical properties suitable for mandibular trabecular bone applications. Cell viability assays confirmed that osteoblastic cells proliferated effectively on the hydroxyapatite scaffolds, and the composite hydrogel successfully induced osteoblast differentiation from undifferentiated mesenchymal stem cells.

The paper “Evaluation of Carboxymethyl Chitosan–Genipin Hydrogels as Reservoir Systems for Suramin Delivery in Epithelial Tissues” by Encinas-Basurto et al. investigates the controlled release of suramin using carboxymethyl chitosan-based hydrogels with varying crosslinking densities (1%, 3%, and 5%). The 1% genipin formulation exhibited the highest drug retention (48.8 ± 6.8 μg/cm^2^ in synthetic membranes; 24.06 ± 7.33 μg/cm^2^ in epithelial models) and enhanced transmembrane flux (>140 μg/cm^2^/h after six hours), demonstrating superior performance for localized treatment applications.

The paper entitled “Polydeoxynucleotide-Loaded Visible Light Photo-Crosslinked Gelatin Methacrylate Hydrogel: Approach to Accelerating Cartilage Regeneration” by Park et al. presents a gelatin methacrylate hydrogel system crosslinked with visible light using riboflavin 5′-phosphate sodium as a photoinitiator. The 14% gelMA-PDRN composition showed optimal performance with sustained PDRN release and enhanced glycosaminoglycan activity. RT-PCR analysis revealed increased expression of cartilage-specific genes (*COL2*, *SOX9*, *AGG*), and histological assessments in a rabbit cartilage defect model demonstrated superior regenerative effects.

The paper “Rapid Multi-Well Evaluation of Assorted Materials for Hydrogel-Assisted Giant Unilamellar Vesicle Production: Empowering Bottom-Up Synthetic Biology” by Tan et al. introduces a controlled drop-casting protocol in multi-well plates for simultaneous evaluation of up to 96 GUV-production formulations. The study successfully evaluated PEG-DA, crosslinked hyaluronic acid, Matrigel, and crosslinked DNA hydrogels, all demonstrating effective GUV production. The protocol enabled successful encapsulation of porcine liver esterase, offering novel GUV labeling capabilities.

The paper entitled “Electrostatic Gelatin Nanoparticles for Biotherapeutic Delivery” by Tobo et al. describes gelatin nanoparticles synthesized via nanoprecipitation with pH adjustments (4.0 or 10.0) to introduce charge variations. Zeta potential measurements validated the electrostatic conjugation of negatively charged mesenchymal stem cell-derived extracellular vesicles with positively charged GNPs. The EV-GNP conjugates demonstrated bioactivity and synergistic effects on macrophage secretory activity over five days of culture.

The paper “Streamlining Skin Regeneration: A Ready-To-Use Silk Bilayer Wound Dressing” by Veiga et al. presents a silk sericin/silk fibroin bilayer construct for wound treatment. The processing methodology included cryopreservation of sericin secondary structure followed by rehydration to produce a hydrogel layer integrated with a salt-leached SF scaffold. The bilayer material exhibited high porosity (>85%) and promoted human dermal fibroblast adhesion, proliferation, and infiltration. A sterilization protocol using supercritical CO_2_ technology was developed for industrial scale-up.

The paper entitled “Coated Microneedle System for Delivery of Clotrimazole in Deep-Skin Mycoses” by Jadach et al. employs 3D printing with photo-curable resin to produce microneedles coated with carbopol-based hydrogels containing clotrimazole. Texture profile analysis demonstrated that ethanol addition significantly affected gel hardness, adhesiveness, and gumminess. Dissolution studies revealed higher clotrimazole release from dissolved-drug formulations, and microbiological testing confirmed efficacy against Candida albicans using both diffusion and suspension-plate methods.

The paper “Investigating the Impact of Mechanical Properties and Cell-Collagen Interaction on NIH3T3 Function: A Comparative Study on Different Substrates and Culture Environments” by Cho and Lee examines fibroblast behavior across three substrate types (non-coated, collagen-coated, and collagen hydrogel) in both 2D monolayer and 3D spheroid cultures. The study demonstrated that 3D spheroid culture maintained fibroblast functionality through enhanced cell–cell interactions, while soft collagen hydrogel substrates provided superior support compared to rigid substrates. Fibroblasts cultured on collagen hydrogel in 2D exhibited comparable functionality to 3D cultures.

The review entitled “The Potential of Functional Hydrogels in Burns Treatment” by Ringrose et al. examines functional hydrogel technologies for burn care applications. The review compares hydrogels to traditional dermal templates, highlighting advantages such as adaptability to irregular wound shapes, moisture retention, controlled drug delivery, and support for cell migration. The analysis addresses structural and biological features influencing performance, including material composition, bioactivity, and integration capacity.

The review “Hydrogels Modulating the Microbiome: Therapies for Tissue Regeneration with Infection Control” by Jiménez-Gastelum et al. explores functionalized hydrogels for microbiome modulation in therapeutic applications. The review discusses the role of skin and gut microbiomes in tissue homeostasis and healing processes. Hydrogel platforms for delivering probiotics (*Lactobacillus plantarum*, *Prevotella histicola*), antimicrobials (silver nanoparticles, nitric oxide donors, bacteriocins), and immune-modulatory agents are examined. The analysis covers innovative approaches including 3D bioprinting, self-healing materials, and photothermal-responsive systems.

## 3. Conclusions and Future Perspectives

The contributions presented in this Special Issue demonstrate the remarkable versatility and therapeutic potential of hydrogels across diverse biomedical applications. The research encompasses fundamental material science investigations, advanced drug delivery systems, tissue-specific engineering approaches, and clinical translation strategies.

Key technological advances highlighted include the development of composite hydrogels with enhanced mechanical properties for bone applications, optimization of crosslinking strategies for controlled drug release, implementation of photo-crosslinking systems for cartilage regeneration, and innovation in delivery platforms for transdermal therapeutic administration.

Future research directions should focus on addressing scalability challenges for clinical translation, developing standardized characterization protocols for regulatory approval, and advancing our understanding of hydrogel–tissue interactions at the molecular level. The integration of advanced manufacturing techniques, including 3D bioprinting and microfluidic fabrication, will enable more sophisticated hydrogel architectures with enhanced functionality.

The interdisciplinary nature of hydrogel research continues to drive innovation through collaboration between materials scientists, biomedical engineers, clinicians, and regulatory specialists. This collaborative approach will be essential for translating promising laboratory results into clinically viable therapies that address unmet medical needs.
